# Carbon Emission Constraint Policy in an OEM and Outsourcing Remanufacturer Supply Chain with Consumer Preferences

**DOI:** 10.3390/ijerph19084653

**Published:** 2022-04-12

**Authors:** Yunting Feng, Yong Geng, Ge Zhao, Mengya Li

**Affiliations:** 1 The Glorious Sun School of Business and Management, Donghua University, Shanghai 200051, China; ytfeng@dhu.edu.cn; 2 School of International and Public Affairs, Shanghai Jiao Tong University, Shanghai 200240, China; ygeng@sjtu.edu.cn; 3 School of Politics and Public Administration, Zhengzhou University, Zhengzhou 450001, China; 4 School of Business, Zhengzhou University, Zhengzhou 450001, China; alexlevi@126.com

**Keywords:** carbon emission constraints, OEM, remanufacturer, outsourcing remanufacturing

## Abstract

Carbon emission reduction has been a consensus goal for most countries to achieve environmental sustainability. The use of carbon emission trading policies has been generally considered by the governments. Remanufacturing, as an effective way to reduce carbon emission, is incorporated together with the tool of carbon emission policy to construct a low-carbon supply chain in this paper. We analyze the carbon emission reduction and profit maximization problem among enterprises of original equipment manufacturers (OEMs) and their outsourcing remanufacturers, integrating the impact of the carbon emission constraint policy and the carbon market. Considering consumer preferences on low-carbon products and recycling rates of waste products, we construct a Stackelberg game model (dominated by the OEM) and analyze the impact of a carbon emission constraint policy on sales price, volume, carbon emission, and revenue of new and remanufactured products in the supply chain system. The results suggest that the upper bound set by the government on carbon emission for enterprises positively affects sales volume of new products and negatively affects sales prices of both products. Moreover, the discount rate of carbon emission constraint negatively affects sales volume of new products and positively affects sales prices of both products. Notably, the carbon emission constraint policy has impacts on the production decisions of both manufacturers on an economic scale. When the upper bound of carbon emission is equal to a certain threshold, the OEM could obtain the greatest revenue. The results provide a new perspective for the government to attain the goal of carbon emission reduction and not sacrifice economic growth. Managers in outsourcing remanufacturers and OEMs could also be implicated from our results to collaborate in allocating remanufacturing orders to achieve win-win opportunities between them.

## 1. Introduction

The Kyoto Protocol came into force in 2005, which motivated the governments around the globe to take effective initiatives to reduce carbon emission. However, global greenhouse gas emissions have reached a historical high level in 2020, and the annual growth rate of carbon emission has been higher than the average level of the past ten years, according to a report by the World Meteorological Organization. Countries are actively increasing the proportion of non-fossil energy consumption, improving energy efficiency, and reducing carbon dioxide emissions [1,2]. China has also reached a commitment in 2020 that it endeavors to achieve the national goal of carbon emission peak by 2030, then to attain carbon emission neutrality by 2060. Other countries are also actively carrying out low-carbon initiatives such as decreasing coal consumption and developing new energy sources [3]. To encourage enterprises to take actions in accordance with the national goals of carbon peak and carbon neutrality, the Chinese government has adopted the carbon cap-and-trade mechanism as one of the common carbon emission reduction policies by imposing a carbon quota on enterprises, in such a way to push enterprises to take carbon reduction actions. Other government initiatives include carbon tax policies [4,5,6,7,8,9,10,11], carbon subsidies [12,13,14,15,16,17,18,19,20,21], take-back regulation [22,23,24], carbon trade market [25,26,27,28,29,30,31,32,33,34], and carbon emission constraints [35,36,37,38,39,40,41,42]. Carbon emission constraint reflected as carbon quota is a policy formulated by the government to limit the excessive carbon emissions of enterprises [42,43,44,45,46]. The government sets an upper bound for carbon emissions which is dependent on an enterprises’ carbon emissions. Exceeding this upper bound is regarded as excessive carbon emissions and will be punished by the government with fines. 

In recent years, remanufacturing has been increasingly addressed by the governments because of its clean production and contribution to carbon reduction [47,48,49,50,51,52,53]. In the production process of new products, some original equipment manufacturers (OEMs) generate excessive carbon emissions due to high energy consumption in manufacturing processes [54]. With regards to this, on one hand, OEMs have to actively adopt various practices such as green manufacturing to reduce their carbon emissions. For instance, Apple established strict objectives and initiatives in 2021 to achieve its goal of Scope 3 carbon neutrality by 2030. Starbucks used paper straws as a substitute for plastic ones. On the other hand, since the OEM’s investment on carbon emission reduction activities is a marginal-effect-decreasing process, consideration beyond internal enterprise for effective emission reduction practices is important. As remanufacturing activities consume significantly less energy and generate products with the same as or close quality to new products, remanufacturers have surplus carbon emission quotas and remanufacturing technologies. In addition, with the increasing environmental consciousness, consumers’ preference for products has moved to remanufactured ones. Hence, OEMs could use an outsourcing strategy to play in the market of remanufacturing as a way of reducing their carbon emission but not reducing their product’s market share [55,56,57]. Such a cooperation network is rarely addressed in the literature but is needed in the setting of carbon emission reduction, with the exploration of the impact of governmental carbon constraint policy. Therefore, our purpose of this study is to construct a sustainable system between OEMs and remanufacturers to optimize the operational factors with consideration of the impact of governmental carbon constraint policy and consumer preference. Through outsourcing remanufacturer recycling waste products, the OEM forms a dual manufacturing/remanufacturing production system to achieve optimization by the integration of resources to reduce carbon emissions and using consumers’ environmental preferences to gain more market shares. OEMs could decide unit sales price, sales volume, and outsourcing cost of new and remanufactured products. However, since new and remanufactured products could replace each other in a functional scale, OEMs need to decide the production results of the two products to maximize the overall profit [33,48]. Therefore, optimizing production strategies based on carbon emission constraint policy is a critical factor faced by OEMs [41,58].

Scholars have studied the impact of carbon emission constraints on the supply chain, for example, research on the impact of carbon emission constraints on supply chains in different markets [37,38,39,40,41,42], how companies decide on the best emission reduction model under low-carbon policies [11,26], and analysis on the production decision-making of the supply chain system based on the joint factors of carbon emissions, quality, capital, and other constraints [59,60,61,62]. Scholars have also studied relevant research on remanufacturing, for example, comparing and analyzing different remanufacturing modes, discussing the boundary conditions of enterprises choosing different remanufacturing modes [63,64,65], the influence of factors such as the enterprise itself and the environment on remanufacturing activities [66,67], based on the supply chain decision model to solve the proportion of outsourcing remanufacturing and the enterprise’s optimal remanufacturing strategy [68,69]. Scholars in the field also investigated the influence of carbon reduction related practices such as life-cycle oriented material selection [70] and renewable energy adoption [71] on the firm performance from the institutional player perspective. Scholars have conducted plentiful research on the remanufacturing model in the context of carbon emission reduction and carbon neutrality, but there is less research on the impact of carbon emission constraints on outsourcing remanufacturing. 

Starting from the research gap, this study develops a two-level supply chain model to explore the effect of carbon emission constraints on the performance outcomes for an OEM and its outsourced remanufacturer. Our research goal is to discover the mechanism of how carbon emission constraining policy by the government takes effects in an outsourcing remanufacturing supply chain system. This article mainly answers the following research questions:

1. How do carbon emission constraints affect optimal production decisions of an OEM and a remanufacturer?
2. What are the economic impacts of carbon emission constraints on an OEM and a remanufacturer? What are the environmental impacts?
3. How does the government set the best carbon emission constraint policy under which the minimal environmental impact and maximum economic performance could be attained in the society?

Using the Stackelberg game analysis method in the established two-level supply chain model for an OEM and a remanufacturer, our research analyzes the boundary conditions and specific effects of the implementation of carbon emission constraint policy on enterprises’ decision-making to achieve optimal profit. Our research develops decision models that involve the critical players of OEMs, remanufacturers, and the government in the carbon reduction process and extends carbon emission literature by enacting carbon constraints as decision variables in the model. We find that the upper bound of carbon emission constraint set by the government is positively correlated with the sales volume of new products, and when the upper bound of carbon emission is equal to a certain threshold, an OEM could achieve maximized profit. The findings provide practical implications for both companies (OEMs and remanufacturers) and the government. An implication for the government is that a suitable carbon constraint policy would stimulate the industry players to get involved in environmentally friendly practices at such an optimal level that minimized environmental impact could be achieved. Industry players could be implicated from our research in the optimal solution of outsourcing remanufacturing, as one of the effective ways to address climate reduction through all stakeholders, establishing a cooperative and win-win governance system.

To answer the research questions, we next introduce the game theory model in Section 2. Section 3 describes the model construction process, and explains the main research conclusions and management implications. Section 4 gives a numerical analysis based on specific research cases of China enterprise. Section 5 summarizes the main findings and discusses them. Section 6 concludes the study and provides future research directions.

## 2. Model Introduction

### 2.1. Problem Description

In an outsourcing remanufacturing system, the profit of a remanufacturer is obtained from outsourced activities, that is, the unit outsourcing price an OEM offers to a remanufacturer for unit remanufactured products, which would directly affect enthusiasm of remanufacturers and the carbon emission reduction based on remanufacturing activities. Therefore, we build the model to analyze the pricing strategy for an OEM on unit new product and remanufactured product and the optimal pricing strategy for a remanufacturer on the outsourcing price of a unit remanufactured product. However, in extant independent remanufacturing models, it is only necessary to analyze the unit price of remanufactured products for a remanufacturer and new products for an OEM. This article considers the manufacturing/remanufacturing supply chain system that is composed of an OEM and a remanufacturer in the context of carbon emission constraint policy. Due to the lack of carbon emission reduction technology and remanufacturing capabilities, the OEMs outsource the remanufacturing activities to the remanufacturers by paying outsourcing costs, and then buy back the remanufactured products and sell both types of products in the market. Meanwhile, the government has formulated relevant carbon emission constraints policies to intervene in the OEM’s production activities, thereby promoting the realization of the carbon emission reduction target. Under this model, in order to maximize their own interests, the OEM decides the unit selling price and sales volume of new and remanufactured products, which indirectly affects the buying decision from the remanufacturer through the outsourcing cost. Therefore, based on the outsourcing remanufacturing model, this article focuses on the constraint boundary of the carbon emission constraint policy on the OEMs and the remanufacturers and further analyzes the OEM’s production decision under the policy.

### 2.2. Model Symbols

Table 1 gives the basic definitions of the symbols used in this article.

### 2.3. Model Function

The demand functions for new and remanufactured products are as below, which are cited from classic literature, i.e., [47,69].

$$p_{n}=1-q_{n}-\delta q_{r},p_{r}=\delta (1-q_{n}-q_{r})$$

According to the literature [47,69], the number of waste products that could be recycled is $q_{r}=\tau q_{n}$, the cost of recycled waste products is $\frac{k}{2}{\left(q_{r}\right)}^{2}$, where k is the coefficient of recycled waste products. 

## 3. Model Establishment and Analysis

### 3.1. Model Establishment

When carbon emission exceeds the government limit, the OEMs will buy carbon quota on the carbon trading market, and the manufacturers with more quotas will sell the remaining carbon quota. The remanufacturer has a greater carbon quota because of the involvement in remanufacturing business, so that a complete carbon trading system will be constructed including the OEM and the remanufacturer. The price of carbon trading plays a vital role in the system, and the costs, benefits, and production decisions of the two manufacturers will change accordingly. This article analyzes the OEM’s decision-making and its impact under the government’s set levels of carbon emission constraints.

(i) Without the carbon emission constraint policy:

(1) $$\mathrm{OEM}:\;\pi_{n}(q_{n},w)=(p_{n}-c_{n})q_{n}+(p_{r}-w)q_{r}$$

(2) $$\mathrm{Remanufacturer}:\;\pi_{r}=(w-c_{r})q_{r}-\frac{k}{2}{\left(q_{r}\right)}^{2}$$

**Lemma** **1.** *(i)* $\pi_{r}$ *of Equation (2) is a concave function with respect to* τ *;*

*(ii) the optimal solution without the carbon emission constraints can be obtained from*

$$\left\{\begin{array}{l} \frac{\partial \pi_{n}^{}}{\partial q_{n}^{}}=1-2q_{n}^{}-c_{n}^{}-\frac{2\delta (w-c_{r}^{})}{k}=0\\ \frac{\partial \pi_{n}^{}}{\partial w}=-\frac{w-c_{r}^{}}{k}-\frac{2\delta q_{n}^{}-\delta +w}{k}-\frac{2\delta (w-c_{r}^{})}{k_{}^{2}}=0 \end{array}\right.$$

(ii) Under the carbon emission constraint policy:

$$e_{n}q_{n}{+e}_{r}\frac{{w-c}_{r}}{k}\ll T$$

(3) $$\mathrm{OEM}:\;\left\{\begin{array}{l} \max\pi_{n}(q_{n},w)=(p_{n}-c_{n})q_{n}+(p_{r}-w)q_{r}\\ s.t.\;e_{n}q_{n}+e_{r}q_{r}=T \end{array}\right.$$

(4) $$\mathrm{Remanufacturer}:\;\pi_{r}=(w-c_{r})q_{r}-\frac{k}{2}{\left(q_{r}\right)}^{2}$$

See Appendix A for the Proof of Lemma 1. According to Equation (A3) in Appendix A, the optimal solution under the carbon emission constraint policy is obtained, as shown in Table 2.

**Conclusion** **1.** *Based on the above model analysis, the optimal solution under the carbon emission constraint policy can be solved:*

**Table 2:** Optimal solutions under the two modes.

Symbol	Without Carbon Emission Constraint Policy (1)	Under Carbon Emission Constraint Policy (2)
$w_{}^{*}$	$\frac{(2\delta^{2}-2\delta -k)c_{r}-k\delta c_{n}}{2(\delta^{2}-\delta -k)}$	$\frac{2k\delta Τe_{n}-2kTe_{r}+ke_{n}e_{r}+4\delta c_{r}e_{n}e_{r}-kc_{n}e_{n}e_{r}-k\delta e_{n}^{2}-2c_{r}e_{r}^{2}-(k+2\delta )c_{r}e_{n}^{2}}{2(2\delta e_{n}e_{r}-ke_{n}^{2}-\delta e_{n}^{2}-e_{r}^{2})}$
$\tau_{}^{*}$	$\frac{c_{r}-\delta c_{n}}{\delta^{2}-\delta -k-\delta c_{r}+(\delta +k)c_{n}}$	$\frac{2\delta Te_{n}-2Te_{r}+e_{n}e_{r}-c_{n}e_{n}e_{r}-\delta e_{n}^{2}+c_{r}e_{n}^{2}}{2\delta Te_{r}-2(k+\delta )Te_{n}+\delta e_{n}e_{r}-c_{r}e_{n}e_{r}-e_{r}^{2}+c_{n}e_{r}^{2}}$
$q_{n}^{*}$	$\frac{1}{2}-\frac{\delta c_{r}-(\delta +k)c_{n}}{2(\delta^{2}-\delta -k)}$	$\frac{2\delta Te_{r}^{}-2(k+\delta )Te_{n}^{}+\delta e_{n}^{}e_{r}^{}-c_{r}^{}e_{n}^{}e_{r}^{}-e_{r}^{2}+c_{n}^{}e_{r}^{2}}{2(2\delta e_{n}^{}e_{r}^{}-ke_{n}^{2}-\delta e_{n}^{2}-e_{r}^{2})}$
$q_{r}^{*}$	$\frac{c_{r}-\delta c_{n}}{2(\delta^{2}-\delta -k)}$	$\frac{2\delta Te_{n}^{}-2Te_{r}^{}+e_{n}^{}e_{r}^{}-c_{n}^{}e_{n}^{}e_{r}^{}-\delta e_{n}^{2}+c_{r}^{}e_{n}^{2}}{2(2\delta e_{n}^{}e_{r}^{}-ke_{n}^{2}-\delta e_{n}^{2}-e_{r}^{2})}$
$p_{n}^{*}$	$\frac{1+c_{n}}{2}$	$\frac{(2\delta +\delta c_{n}^{}+c_{r}^{})e_{n}^{}e_{r}^{}+(-2k-2\delta +\delta^{2}-\delta c_{r}^{})e_{n}^{2}+2(k+\delta -\delta^{2})Te_{n}^{}-(1+c_{n}^{})e_{r}^{2}}{2(2\delta e_{n}^{}e_{r}^{}-ke_{n}^{2}-\delta e_{n}^{2}-e_{r}^{2})}$
$p_{r}^{*}$	$\delta \left[\frac{1}{2}+\frac{\delta c_{r}-c_{r}-kc_{n}}{2(\delta^{2}-\delta -k)}\right]$	$\frac{3\delta^{2}e_{n}^{}e_{r}^{}+(c_{n}^{}+c_{r}^{}-1)\delta e_{n}^{}e_{r}^{}-(\delta +2k+c_{r}^{})\delta e_{n}^{2}-(1+c_{n}^{})\delta e_{r}^{2}+2(1-\delta )\delta Te_{r}^{}+2k\delta Te_{n}^{}}{2(2\delta e_{n}^{}e_{r}^{}-ke_{n}^{2}-\delta e_{n}^{2}-e_{r}^{2})}$

### 3.2. Model Analysis

**Conclusion** **2.** *The impact of carbon emission constraints on the environment:*

 *(i)*  *Without the carbon emission constraint policy, the total carbon emission of new products is*$E_{n1}=e_{n}q_{n1}^{*}=\frac{e_{n}}{2}-\frac{\delta c_{r}e_{n}-(\delta +k)c_{n}e_{n}}{2(\delta^{2}-\delta -k)}$, *the total carbon emission of remanufactured products is*$E_{r1}=e_{r}q_{r1}^{*}=\frac{c_{r}e_{r}-\delta c_{n}e_{r}}{2(\delta^{2}-\delta -k)}$, *and the total carbon emission of two types of products is*$E_{1}=\frac{e_{n}}{2}+\frac{(c_{r}-\delta c_{n})e_{r}+\left[(k+\delta )c_{n}-\delta c_{r}\right]e_{n}}{2(\delta^{2}-\delta -k)}$.
 *(ii)*  *Under the carbon emission constraint policy, the total carbon emission of new products is *$E_{n2}=e_{n}q_{n2}^{*}=\frac{2\delta Te_{n}e_{r}^{}-2(k+\delta )Te_{n}^{2}+\delta e_{n}^{2}e_{r}^{}-c_{r}^{}e_{n}^{2}e_{r}^{}-e_{n}e_{r}^{2}+c_{n}^{}e_{n}e_{r}^{2}}{2(2\delta e_{n}^{}e_{r}^{}-ke_{n}^{2}-\delta e_{n}^{2}-e_{r}^{2})}$*, the total carbon emission of remanufactured products is*$E_{r2}=e_{r}q_{r2}^{*}=\frac{2\delta Te_{n}^{}e_{r}-2Te_{r}^{2}+e_{n}^{}e_{r}^{2}-c_{n}^{}e_{n}^{}e_{r}^{2}-\delta e_{n}^{2}e_{r}+c_{r}^{}e_{n}^{2}e_{r}}{2(2\delta e_{n}^{}e_{r}^{}-ke_{n}^{2}-\delta e_{n}^{2}-e_{r}^{2})}$*, and the total carbon emission of two types of products is*$E_{2}=T$.

Conclusion 2 shows that when $E_{2}\geq E_{1}^{}$, that is $T\geq \frac{e_{n}}{2}+\frac{(c_{r}-\delta c_{n})e_{r}+\left[(k+\delta )c_{n}-\delta c_{r}\right]e_{n}}{2(\delta^{2}-\delta -k)}$, the total carbon emission of two types of products when both the OEM and remanufacturer produce based on the optimal decision-making points is less than or equal to the upper bound of carbon emission set by the government. The carbon emission constraint has no impact on the production decisions of the two manufacturers. When $E_{2}<E_{1}^{}$, that is $T<\frac{e_{n}}{2}+\frac{(c_{r}-\delta c_{n})e_{r}+\left[(k+\delta )c_{n}-\delta c_{r}\right]e_{n}}{2(\delta^{2}-\delta -k)}$, the total carbon emission of the two manufacturers when they produce based on the optimal decision-making points is greater than the carbon emission upper bound set by the government. The carbon emission constraint policy will affect the production decisions of the two manufacturers, and the production activities that exceed the carbon emission upper bound will be punished by the government.

According to Conclusion 2, when the policy of carbon emission constraint E2 is greater or equal to E1, it will not affect the production decision of the two manufacturers. In this case, it is meaningless to implement the carbon emission constraint policy. Therefore, the following assumptions need to be given.

**Assumption.**  *The value range of the carbon emission constraint should meet* $T<\frac{e_{n}}{2}+\frac{(c_{r}-\delta c_{n})e_{r}+\left[(k+\delta )c_{n}-\delta c_{r}\right]e_{n}}{2(\delta^{2}-\delta -k)}$ *, otherwise, the implementation of the carbon emission constraint policy will not have an impact on the original production behavior of the two manufacturers.*

Corollary 1 can be derived from Conclusion 2, which is as follows:

**Corollary** **1.** *Under the carbon emission constraint policy, the environmental friendliness of new products and remanufacturing is as follows:*

*When*$\delta >\frac{kTe_{n}^{2}+Te_{r}^{2}-e_{n}e_{r}^{2}-c_{r}e_{n}^{2}e_{r}^{}+c_{n}e_{n}e_{r}^{2}}{e_{n}^{2}(e_{r}-T)}$, $E_{n2}>E_{r2}$*, otherwise,*$E_{n2}\leq E_{r2}$.

*According to Corollary 1, under the carbon emission constraint policy, remanufactured products are not always environmentally friendly. It depends on consumers’ preference for new and remanufactured products and the upper bound of carbon emissions. Only when the product preference is large enough and the government sets a reasonable upper bound on carbon emissions can the carbon emission of remanufactured products be lower than that of new products.*

**Conclusion** **3.** *Under the constraint of carbon emissions, the impact of the upper bound of carbon emission set by the government on unit outsourcing cost, unit sales prices, and sales volume is as follows:*

 *(i)*  $\frac{\partial w_{2}^{*}}{\partial T}=\frac{k(\delta e_{n}-e_{r})}{2\delta e_{n}e_{r}-ke_{n}^{2}-\delta e_{n}^{2}-e_{r}^{2}}$*, when*$\delta >\frac{e_{r}}{e_{n}}$, $\frac{\partial w_{2}^{*}}{\partial T}<0$*, otherwise,*$\frac{\partial w_{2}^{*}}{\partial T}\geq 0$;
 *(ii)*  $\frac{\partial q_{n2}^{*}}{\partial T}=\frac{\delta e_{r}-(k+\delta )e_{n}}{2\delta e_{n}e_{r}-ke_{n}^{2}-\delta e_{n}^{2}-e_{r}^{2}}>0$; $\frac{\partial q_{r2}^{*}}{\partial T}=\frac{\delta e_{n}-e_{r}}{2\delta e_{n}e_{r}-ke_{n}^{2}-\delta e_{n}^{2}-e_{r}^{2}}$*, when*$\delta >\frac{e_{r}}{e_{n}}$, $\frac{\partial q_{r2}^{*}}{\partial T}<0$*, otherwise,*$\frac{\partial q_{r2}^{*}}{\partial T}\geq 0$;
 *(iii)*  $\frac{\partial p_{n2}^{*}}{\partial T}=\frac{(k+\delta -\delta^{2})e_{n}}{2\delta e_{n}e_{r}-ke_{n}^{2}-\delta e_{n}^{2}-e_{r}^{2}}<0$; $\frac{\partial p_{r2}^{*}}{\partial T}=\frac{\delta (1-\delta )e_{r}+k\delta e_{n}}{2\delta e_{n}e_{r}-ke_{n}^{2}-\delta e_{n}^{2}-e_{r}^{2}}<0$.

According to Conclusion 3, an increase in the upper bound of carbon emission can reduce the unit sales prices of two types of products and increase the sales volume of new products. However, the upper bound of carbon emission is not always positively correlated with the sales volume of remanufactured products and the unit outsourcing cost. It requires the sales prices of the two products and the unit carbon emission of the two products to meet certain conditions. Only when $\delta <\frac{e_{r}}{e_{n}}$ is attained can an increase in the upper bound of carbon emission increase the sales volume of remanufactured products and increase the unit outsourcing cost.

Similar to [43,44], under the carbon emission constraint, manufacturers’ production decisions are related to the upper bound of carbon emission and would be affected by the ratio of sales prices of the two products and the ration of carbon emission of unit two products. For the OEMs, when the upper bound of carbon emission increases, the production number of new products increases. To obtain more profit, OEMs will reduce unit sales prices to stimulate consumers to purchase more new products. Moreover, the unit sales price of remanufactured products will decrease as the upper bound of carbon emission increases. In addition, as a competitor of new products, the production number of remanufactured products will decrease as more consumers prefer new products with pricing down. When the ratio of the sales price of unit remanufactured product to that of new product is less than the ratio of unit carbon emission of the two products, the unit outsourcing cost that the OEM is willing to pay and the sales volume of remanufactured products will be in line with the upper bound of carbon emission. The optimal production number of remanufactured products is related to unit outsourcing cost and unit production cost. The increase in unit outsourcing cost will encourage remanufacturers to find ways to increase the recycling rate of waste products and increase the production number [45]. OEMs make production decisions based on consumer preferences, carbon emission policy [72,73], and unit outsourcing cost and affect the production number of remanufactured products. In doing so, OEMs could coordinate the market share of the two products to maximize their own revenues.

**Management** **Enlightenment** **1.** *Under the constraint of carbon emission policy, OEMs will adjust production number and sales prices in response to the adjustment of the upper bound of carbon emission, which will affect production number of remanufactured products by adjusting unit outsourcing cost. Remanufacturers will determine the optimal production number and recycling rate of waste products based on the outsourcing cost, production cost, and carbon emission. Therefore, changes in the upper bound of carbon emission will affect the production behavior of two manufacturers and indirectly affect consumer behavior. To better achieve low-carbon production, the government can take measures to directly guide consumer preferences, such as promoting and educating consumers to choose remanufactured products.*

**Conclusion** **4.** *The impacts of carbon emission constraint policies on unit sales price, unit outsourcing cost, and sales volume of two products are as follows:*

 *(i)*  $p_{n2}^{*}>p_{n1}^{*}$; $p_{r2}^{*}>p_{r1}^{*}$;
 *(ii)*  *When*$\delta <\frac{e_{r}}{e_{n}}$, $w_{2}^{*}<w_{1}^{*}$*, otherwise,*$w_{2}^{*}\geq w_{1}^{*}$;
 *(iii)*  $q_{n2}^{*}<q_{n1}^{*}$;
  *when*$\delta <\frac{e_{r}}{e_{n}}$, $q_{r2}^{*}<q_{r1}^{*}$*, otherwise,*$q_{r2}^{*}\geq q_{r1}^{*}$.

See Appendix A for the Proof of Conclusion 4. According to Conclusion 4, the sales volume of new products is reduced under the emission restriction policy. Carbon emission policy restricts the use of carbon quotas by the two manufacturers. The per unit carbon emission of new products is greater than that of remanufactured products and the production number of new products will be bounded. In this case, OEMs will choose to increase outsourcing cost to encourage remanufacturers to produce more or to increase unit new product sales price to compensate for the loss caused by reduced production number. Since the two products are competitive, the increase in remanufacturing production and sales price of new products will reduce the sales volume of new products.

Similar to [73], Considering when the discount rate of the carbon emission constraint policy is greater than the ratio of the carbon emission of the two products, the unit outsourcing cost under carbon emission constraint is greater than that without the constraint. As consumers’ awareness of environmental protection increases and consumers prefer to purchase remanufactured products, OEMs will encourage remanufacturers to increase production by increasing unit outsourcing cost [72]. However, the increase in outsourcing cost in this case leads to an increase in costs of OEMs, which leads to the OEM increasing unit sales prices of both products. Another setting is when the discount rate of the carbon emission is less than the ratio of the carbon emission of two products, the unit outsourcing cost with carbon constraint will be less than that without carbon constraint. Due to insufficient consumer preference, the consumption of remanufactured products is not ideal. Therefore, OEMs will not increase unit outsourcing cost. In this case, OEMs will increase the sales prices of new and remanufactured products to compensate the loss caused by the decrease in sales volume.

**Management** **Enlightenment** **2.** *The carbon emission constraint policy can limit the output of new products with higher carbon emissions, but at the same time, it will also increase the sales prices of both products, and whether the production number of remanufactured products increases depends on the ratio of the discount rate of carbon emission constraint. To carry out low carbon production, the government should continue to find ways to increase consumers’ environmental preferences and promote the production and sales of remanufactured products. As the sales of remanufactured products increases, remanufacturing technology research and development expenses and marketing costs will increase, which would further encourage the development of the remanufacturing industry [44]. In the long run, this policy will effectively reduce production costs and recycling costs of remanufacturing and reduce carbon emissions, forming a greater and more sustainable mode for remanufacturing industry development.*

**Conclusion** **5.** *The impact of carbon emission constraint policy on the revenue of the two manufacturers is as follows:*

$$M=-8\delta_{}^{2}c_{r}^{}-8\delta_{}^{2}+8\delta_{}^{3}-8k\delta +12\delta_{}^{2}c_{n}^{}+12k\delta c_{n}^{}-4\delta c_{r}^{}-4kc_{r}^{}$$

$$N=-8\delta_{}^{2}c_{n}^{}+12\delta c_{r}^{}-4kc_{n}^{}-4\delta c_{n}^{}-4\delta_{}^{2}+4\delta +4k$$

$$V=4k_{}^{2}c_{n}^{}+8k\delta c_{n}^{}-4k_{}^{2}-8k\delta -4k\delta c_{r}^{}+4\delta_{}^{2}(k+c_{n}^{}-1+\delta -c_{r}^{})$$

 *(i)*  *When*$T<\frac{Ve_{n}^{3}+4(c_{r}^{}-\delta c_{n}^{})e_{r}^{3}-Me_{n}^{2}e_{r}^{}-Ne_{n}^{}e_{r}^{2}}{8(k+\delta -\delta_{}^{2})(2\delta e_{n}^{}e_{r}^{}-ke_{n}^{2}-\delta e_{n}^{2}-e_{r}^{2})}$, $\frac{\partial \pi_{n2}^{*}}{\partial T}>0$*, otherwise,*$\frac{\partial \pi_{n2}^{*}}{\partial T}\leq 0$.
 *(ii)*  *When*$T<\frac{-2k\left[(2\delta -\delta c_{n}^{}-c_{r}^{})e_{n}^{2}e_{r}^{}+(c_{n}^{}-1)e_{r}^{2}e_{n}^{}+(\delta c_{r}^{}-\delta_{}^{2})e_{n}^{3}\right]}{4k{\left(\delta e_{n}^{}-e_{r}^{}\right)}_{}^{2}}$, $\frac{\partial \pi_{r2}^{*}}{\partial T}<0$*, otherwise,*$\frac{\partial \pi_{r2}^{*}}{\partial T}\geq 0$

See Appendix A for the Proof of Conclusion 5. According to Conclusion 5, carbon emission constraint will increase the sales price of both products, but it will not always increase the revenues of the two manufacturers. When the upper bound of carbon emission is less than the threshold as indicated in (*i*), the OEM’s revenue is positively related to the upper bound of carbon emission. When the upper bound of carbon emission is greater than the threshold and less than the constraint value of the assumptions as indicated in (*ii*), its revenue is negatively related to the upper bound of carbon emission, which is not conducive for OEMs to increase their revenue [42]. In addition, when the upper bound of carbon emission is lower than the threshold, it will greatly restrict the manufacturer’s production. Increase in the upper bound can significantly increase production, and revenue from the increased production can compensate for the loss caused by the decrease in unit sales price of the product, thereby increasing revenue of OEMs. When the upper bound of carbon emission is greater than the threshold, it will have no significant impact on the manufacturer’s production activities [40]. Increase in the upper bound will not significantly increase production, and the increased revenue by increased production will not compensate for the loss caused by the reduction in unit sales price. It can be seen that only when the upper bound of carbon emission constraint is equal to this threshold, OEMs’ benefit arrives at the largest level.

Similar to [52,57,58], when the upper bound of carbon emission is less than the threshold, revenue of the remanufacturer is negatively correlated with the bound. When the bound is greater than the threshold and less than the assumed constraint, revenue of the remanufacturer is positively correlated with the bound. When the bound is lower than the threshold and the discount rate is greater than the ratio of the carbon emissions of both products, an increase in bound will lead to a reduction in unit outsourcing cost and sales volume of remanufactured products, which will result in a reduction in the revenue of remanufacturers. When the bound is lower than the threshold and the discount rate is less than the ratio of the carbon emissions of both products, an increase in the bound will lead to an increase in unit outsourcing cost and the sales volume of remanufactured products. However, the loss is greater than the benefit brought by the reduction of recycling costs; thus, reduction in the revenue of remanufacturers will happen. When the upper bound is greater than the threshold and the discount rate is greater than the ratio of the carbon emissions of both products, an increase in the bound will decrease unit outsourcing cost and sales volume of remanufactured products, but the decrease would lead to an increase in outsourcing costs, which will increase the revenue of remanufacturers. When the upper bound is greater than the threshold, if the discount rate is less than the ratio of the carbon emissions of both products, an increase in the bound will lead to an increase in unit outsourcing costs and the sales volume of remanufactured products, and the loss is less than the benefit brought by the reduction of recycling costs, which will increase the revenue of remanufacturers. It can be seen that remanufacturers should not only adjust production activities according to the upper limit of carbon emissions but also upgrade their own production technology and increase the recycling rate of waste products.

**Management** **Enlightenment** **3.** *Under carbon emission constraint policy, two manufacturers will adjust their production activities according to the upper bound of carbon emission to maximize revenues. OEMs make production decisions by adjusting production number, unit sales price, and unit outsourcing cost; remanufacturers make production decisions based on unit outsourcing cost and consumer preferences. Therefore, if the upper bound set by the government is too low, it will significantly affect the production enthusiasm of the two manufacturers. The government should fully consider the production decisions and revenues of two manufacturers under different carbon emission bounds. In doing so, an effective carbon emission constraint policy should contribute to reduction of total carbon emission.*

## 4. Numerical Analysis

To specifically study the impact of carbon emission constraint policy on the unit sales price, sales volume, and revenue of both products under outsourcing remanufacturing strategy, this article takes the case of automobile engine remanufacturing in China as an example to carry out a numerical analysis. According to research on the Chinese remanufacturing industry [69], a remanufactured product averagely reduces 60% more pollution during its remanufacturing process compared to the manufacturing process of a same and new product. Therefore, we set $e_{n}^{}=1$, $e_{r}^{}=0.4$. Moreover, the average cost of producing a remanufactured product in China is 50% or less than that of a new product [69], so we set $c_{n}^{}=0.2$, $c_{r}^{}=0.1$, k=1.1, which are also cited by [72].

### 4.1. The Impact of T and δ on Unit Sales Prices of New Products and Remanufactured Products

**Figure 1:**
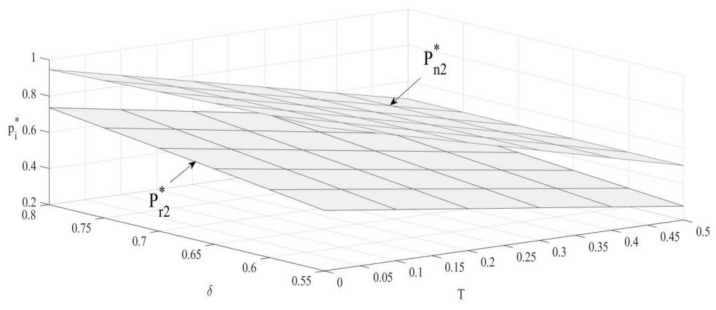
The impact of T and δ on unit sales prices.

From Figure 1, the unit sales prices of both products are negatively correlated with the upper bound of carbon emission. When the carbon emission bound set by the government is raised up, the production number of new products will increase, and the OEM will increase sales volume through promotional strategies such as pricing down [35]. As the competitor, the remanufacturer will also decrease sales price to avoid competitive disadvantages.

**Corollary** **2.** *The impacts of consumer preference on unit sales prices of new and remanufactured products are:*

$$\frac{\partial p_{n2}^{*}}{\partial \delta }<0;\;\frac{\partial p_{r2}^{*}}{\partial \delta }>0$$

Unit sales price of new products is negatively correlated with consumer preference, while unit sales price of remanufactured products is positively correlated with consumer preference. Under the carbon emission constraint policy, remanufactured products with emission advantage will increase consumers’ purchasing attention. As consumers’ preference for remanufactured products increases, OEMs will increase the sales price of remanufactured products to obtain higher revenue. Meanwhile, OEMs will reduce the sales price of new products to avoid a substantial decrease in sales.

### 4.2. The Impact of T and δ on Sales Volume of New Products and Remanufactured Products

**Figure 2:**
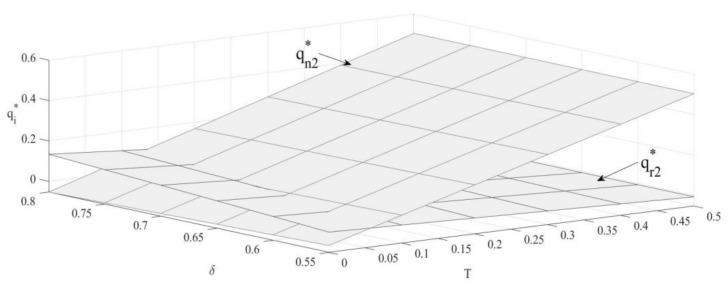
The impact of T and δ on sales volume.

From Figure 2, sales volume of new products is positively correlated with the upper bound of emission and that of remanufactured products is negatively correlated with the bound. Since remanufactured products with emission advantages will be more attractive to consumers [50,51], OEMs will reduce the production of new products and encourage remanufacturers to produce more by increasing the unit outsourcing cost.

**Corollary** **3.** *The impacts of consumer preference on the sales volume of new and remanufactured products are:*

$$\frac{\partial q_{n2}^{*}}{\partial \delta }<0;\;\frac{\partial q_{r2}^{*}}{\partial \delta }>0$$

Sales volume of new products is negatively correlated with consumer preference, while sales volume of remanufactured products is positively correlated with consumer preference. Under the carbon emission constraint policy, as consumer preference for remanufactured products increases, OEMs will encourage remanufacturers to produce more, which increases sales volume of remanufactured products and decreases that of new products.

### 4.3. The Impact of T and δ on the Revenue of the OEM and Remanufacturer

**Figure 3:**
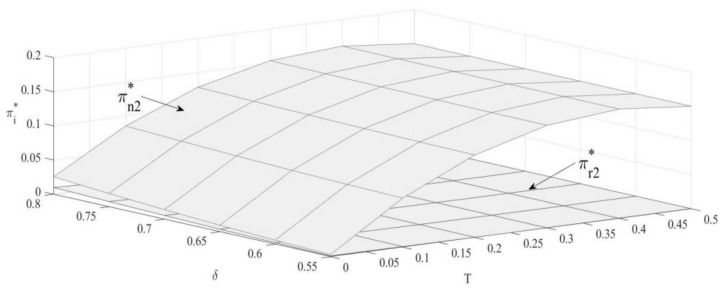
The impact of T and δ on revenue.

From Figure 3, as the upper bound of carbon emission increases, revenue of the OEMs shows a non-linear trend: increasing firstly and then decreasing; additionally, revenue of remanufacturers shows an opposite non-linear relation: decreasing firstly and then increasing, which is in accordance with [39]. When the upper bound of carbon emission is small, it will impose greater restrictions on manufacturer’s production. Raising up the upper bound can effectively increase production number, thereby increasing the OEM’s revenue. Meanwhile, raising up the upper bound will reduce unit outsourcing cost and the sales volume of remanufactured products. When the bound is low, production number of remanufactured products is more sensitive to changes of bound value. Moreover, the loss caused by unit outsourcing cost reduction and sales volume reduction of remanufactured products is not large enough to cover the outsourcing cost increasing, so the revenue of the remanufacturer is reduced.

**Corollary** **4.** *The impacts of consumer preferences on the revenue of the OEM and remanufacturer are:*

$$\frac{\partial \pi_{n2}^{*}}{\partial \delta }>0;\;\frac{\partial \pi_{r2}^{*}}{\partial \delta }>0$$

Similar to [73,74], our analysis reveals that revenues of both manufacturers positively relate to consumer preference. Under the carbon emission constraint policy, as consumer preference for remanufactured products increases, the demand will increase, and it further increases the production. Therefore, revenue of remanufacturers increases. Meanwhile, OEMs also increase their revenues as the demand of remanufactured products increases.

## 5. Discussion

By comparing the optimal solutions for the OEM and the remanufacturer with and without the government policy carbon emission reduction, we extend the literature in the following aspects.

First, the upper bound of carbon emission constraint set by the government is positively correlated with the sales volume of new products and negatively correlated with either the price of new products or that of remanufactured ones. Such a result is in accordance with Yenipazarli [75]. It indicates that a stricter carbon emission policy (with a small upper bound value) would do harm to the new products market regarding price and sales volume.

Second, we also extend this finding by analyzing the impact of a discount rate of carbon constraint. It proves that if the discount rate is greater than the ratio of carbon emission of remanufactured products to new products, the upper bound will negatively correlate with unit outsourcing cost and the sales volume of remanufactured products. According to the literature [76], consumer preference would affect the relationship between low-carbon practices performance. We extend the literature and prove that the government should set an upper bound of carbon emission to such a level that maximizes the sales volume of remanufactured products.

Third, carbon emission contraint policy could significantly affect the production decisions of both manufacturers in the industry. When the upper bound of carbon emission is equal to a certain threshold, an OEM could achieve maximized profit. It indicates that the government should not neglect the situation of both manufacturers when determining the upper bound of carbon emission constraint, so as to achieve balance between economic goals and carbon concerns.

## 6. Conclusions

By constructing a game model between an OEM and a remanufacturer based on the competition mechanism of two manufacturers, this work analyzes the impact of carbon emission constraints on the optimal outsourcing decisions of the OEM outsourcing. Our research enriches the literature in the field by developing decision models that involve the critical players of OEMs, remanufacturers, and the government in the carbon reduction process and extends carbon emission literature by enacting carbon constraints as a decision variable in the model. The results implicate that outsourcing remanufacturing is one effective way to address climate reduction through all stakeholders establishing a cooperative and win-win governance system, but the premise of which, as we have proved, is using a grounded and precise carbon emission constraint policy to assure the positive influence on manufacturing industries economically. Moreover, our analysis contributes to the practitioners by suggesting that additional tools used by the government to improve consumer preference in remanufactured products such as promotion is needed. To better achieve the goal of carbon peak as well as carbon neutrality, the government should promote the remanufacturing industries by legislating more practical guidelines such as cooperation between remanufacturers and OEMS.

Since our research focuses on outsourcing remanufacturing, future research could add the impact of recycling propaganda on the manufacturing/remanufacturing into the model. Another direction is to further study the impact of different carbon emission constrained price mechanisms on the remanufacturing industry based on the international trend of carbon trading policies.

## Figures and Tables

**Table 1:** Definition of symbols.

Symbol	Definition
n,r	OEM, remanufacturer;
$q_{n},q_{r}$	Sales volume of new and remanufactured products;
$p_{n},p_{r}$	Unit sales prices of new and remanufactured products;
$c_{n},c_{r}$	Unit production cost of new and remanufactured products (in reality, it is known that $c_{n}>c_{r}$);
Subscript 1,2	The optimal solution under the carbon emission constraint policy; the optimal solution without the carbon emission constraint policy;
T	The upper bound of carbon emission by OEMs and remanufacturers;
$e_{n},e_{r}$	The carbon emissions of unit new product and unit remanufactured product (that is, the environmental impact of unit new product and unit remanufactured product; in reality, it is known that $e_{n}>e_{r}$);
$E_{n},E_{r}$	The total carbon emissions of new products and remanufactured products, that is $E_{n}=e_{n}q_{n}$, $E_{r}=e_{r}q_{r}$;
E	The total carbon emissions of both products, that is, the total impact of the two manufacturers’ production on the environment;
τ	The ratio of the number of waste products recycled by remanufacturers to the sales volume of new products by OEMs (that is, the recycling rate of waste products);
δ	The ratio of the sales price of unit remanufactured product to the sales price of unit new product, which indicates the consumer’s preference for remanufactured products (in reality, it is known that 0≤δ≤1);
w	The outsourcing cost of unit remanufactured product paid by an OEM to the remanufacturer;
$\pi_{i}(i=n,r)$	The revenue earned by the OEMs;
$\frac{k}{2}{\left(q_{r}\right)}^{2}$	Waste product recycling cost, where *k* is the coefficient of recycling waste products.

## Data Availability

The data come from a medium-sized used engine remanufacturing firm in China, Jinan Fuqiang Company.

## Appendix A

**Proof** **of** **Lemma** **1.** (i) Bringing $q_{r}=\tau q_{n}$ into Equation (2) in the manuscript, we can get:

(A1) $$\pi_{r}=(w-c_{r})\tau q_{n}-\frac{k}{2}{\left(\tau q_{n}\right)}^{2}$$

The first-order partial derivative and the second-order partial derivative of τ on *π_r_* in Equation (A1) are as follows:

$$\frac{\partial \pi_{r}}{\partial \tau }=(w-c_{r})q_{n}-k\tau q_{n}^{2}$$

$$\frac{\partial^{2}\pi_{r}}{\partial \tau^{2}}=-kq_{n}^{2}$$

It can be seen from $\frac{\partial^{2}\pi_{r}}{\partial \tau^{2}}=-kq_{n}^{2}<0$ that Equation (2) in the manuscript is a concave function of τ; By setting the first derivative of τ on *π_r_* in Equation (A1) equal to 0, we can get: $\tau^{*}=\frac{w-c_{r}}{kq_{n}}$. Therefore, the remanufacturer’s waste product recovery rate and profit can be obtained by solving the maximum value of the objective function of the OEM. Bringing $p_{n}=1-q_{n}-\delta q_{r}$, $p_{r}=\delta (1-q_{n}-q_{r})$, $q_{r}=\tau^{*}q_{n}$ into Equation (1) in the manuscript:

(A2) $$\pi_{n}(q_{n},w)=(1-q_{n}-\delta \frac{w-c_{r}}{k}-c_{n})q_{n}+\left[\delta (1-q_{n}-\frac{w-c_{r}}{k})-w\right]\frac{w-c_{r}}{k}$$

The Hessian matrix can be obtained from the objective profit function of the OEMs $H=\left[\begin{array}{cc} -2 & -\frac{2\delta }{k}\\ -\frac{2\delta }{k} & -\frac{2}{k}-\frac{2\delta }{k^{2}} \end{array}\right]$, $|H|=\frac{4}{k^{2}}\left[k+\delta (1-\delta )\right]>0$, and −2<0, therefore, the objective profit function of the OEM is a concave function of $q_{n}$, w, and there is an optimal solution. According to the theory of nonlinear programming, this article introduces the generalized Lagrangian factor λ, according to Equation (A2), the OEM’s revenue function is as follows:

$$L=(1-q_{n}-\delta \frac{w-c_{r}}{k}-c_{n})q_{n}+\left[\delta (1-q_{n}-\frac{w-c_{r}}{k})-w\right]\frac{w-c_{r}}{k}+\lambda (T-e_{n}^{}q_{n}^{}-e_{r}^{}\frac{w-c_{r}^{}}{k})$$

Set the *K*-*T* point is $q_{n}^{*}$, $w_{}^{*}$, then the *K*-*T* condition is as follows:

(A3) $$\left\{\begin{array}{l} 1-2q_{n}^{}-c_{n}^{}-\frac{2\delta (w-c_{r}^{})}{k}-\lambda e_{n}^{}=0\\ \frac{-2\delta q_{n}^{}+\delta -2w+c_{r}^{}}{k}-\frac{2\delta (w-c_{r}^{})}{k_{}^{2}}-\frac{\lambda e_{r}^{}}{k}=0\\ \lambda \left[T-e_{n}^{}q_{n}^{}-e_{r}^{}\frac{\left(w-c_{r}^{}\right)}{k}\right]=0 \end{array}\right.$$

□

**Proof** **of** **Conclusion** **4.** (i) According to Conclusion 1:

$$p_{n1}^{*}=\frac{1+c_{n}}{2}$$

$$p_{n2}^{*}=\frac{(2\delta +\delta c_{n}^{}+c_{r}^{})e_{n}^{}e_{r}^{}+(-2k-2\delta +\delta^{2}-\delta c_{r}^{})e_{n}^{2}+2(k+\delta -\delta^{2})Te_{n}^{}-(1+c_{n}^{})e_{r}^{2}}{2(2\delta e_{n}^{}e_{r}^{}-ke_{n}^{2}-\delta e_{n}^{2}-e_{r}^{2})}$$

$$p_{n2}^{*}-p_{n1}^{*}=\frac{c_{r}^{}e_{n}^{}e_{r}^{}-\delta c_{n}^{}e_{n}^{}e_{r}^{}-\delta c_{r}^{}e_{n}^{2}+(k+\delta )c_{n}^{}e_{n}^{2}+(\delta^{2}-\delta -k)e_{n}^{2}-2(\delta^{2}-\delta -k)Te_{n}^{}}{2(2\delta e_{n}^{}e_{r}^{}-ke_{n}^{2}-\delta e_{n}^{2}-e_{r}^{2})}$$

$T<\frac{e_{n}}{2}+\frac{(c_{r}-\delta c_{n})e_{r}+\left[(k+\delta )c_{n}-\delta c_{r}\right]e_{n}}{2(\delta^{2}-\delta -k)}$, $p_{n2}^{*}-p_{n1}^{*}>0$, that is $p_{n2}^{*}>p_{n1}^{*}$. (i) is proven. (ii) and (iii) can be proven similarly. The proof of Conclusion 4 is completed. □

**Proof** **of** **Conclusion** **5.** According to Conclusion 1:

(A4) $$\pi_{n2}^{*}=(p_{n2}^{*}-c_{n})q_{n2}^{*}+(p_{r2}^{*}-w_{2}^{*})q_{r2}^{*}$$

$$\begin{array}{l} \frac{\partial \pi_{n2}^{*}}{\partial T}=\frac{\partial (p_{n2}^{*}-c_{n})}{\partial T}q_{n2}^{*}+\frac{\partial q_{n2}^{*}}{\partial T}(p_{n2}^{*}-c_{n})+\frac{\partial (p_{r2}^{*}-w_{2}^{*})}{\partial T}q_{r2}^{*}+\frac{\partial q_{r2}^{*}}{\partial T}(p_{r2}^{*}-w_{2}^{*}) \end{array}$$

Substituting $p_{n2}^{*}$, $p_{r2}^{*}$, $q_{n2}^{*}$, $q_{r2}^{*}$, $w_{2}^{*}$ obtained in Conclusion 1 into the above equation, we can get:

$$\frac{\partial \pi_{n2}^{*}}{\partial T}=\frac{8T(k+\delta -\delta_{}^{2})(2\delta e_{n}^{}e_{r}^{}-ke_{n}^{2}-\delta e_{n}^{2}-e_{r}^{2})-Ve_{n}^{3}-4(c_{r}^{}-\delta c_{n}^{})e_{r}^{3}+Me_{n}^{2}e_{r}^{}+Ne_{n}^{}e_{r}^{2}}{{\left[2(2\delta e_{n}^{}e_{r}^{}-ke_{n}^{2}-\delta e_{n}^{2}-e_{r}^{2})\right]}_{}^{2}}$$

From $\frac{\partial_{}^{2}\pi_{n2}^{*}}{\partial T_{}^{2}}=\frac{8(k+\delta -\delta_{}^{2})(2\delta e_{n}^{}e_{r}^{}-ke_{n}^{2}-\delta e_{n}^{2}-e_{r}^{2})}{{\left[2(2\delta e_{n}^{}e_{r}^{}-ke_{n}^{2}-\delta e_{n}^{2}-e_{r}^{2})\right]}_{}^{2}}<0$, Equation (A4) is a concave function, and the optimal solution of the function is $T=\frac{Ve_{n}^{3}+4(c_{r}^{}-\delta c_{n}^{})e_{r}^{3}-Me_{n}^{2}e_{r}^{}-Ne_{n}^{}e_{r}^{2}}{8(k+\delta -\delta_{}^{2})(2\delta e_{n}^{}e_{r}^{}-ke_{n}^{2}-\delta e_{n}^{2}-e_{r}^{2})}$. Thus, when $T<\frac{Ve_{n}^{3}+4(c_{r}^{}-\delta c_{n}^{})e_{r}^{3}-Me_{n}^{2}e_{r}^{}-Ne_{n}^{}e_{r}^{2}}{8(k+\delta -\delta_{}^{2})(2\delta e_{n}^{}e_{r}^{}-ke_{n}^{2}-\delta e_{n}^{2}-e_{r}^{2})}$, $\frac{\partial \pi_{n2}^{*}}{\partial T}>0$, otherwise, $\frac{\partial \pi_{n2}^{*}}{\partial T}\leq 0$. (i) is proven. (ii) can be proven similarly. The proof of Conclusion 5 is completed. □
